# Platelet-Adherent Leukocytes Associated With Cutaneous Cross-Reactive Hypersensitivity to Nonsteroidal Anti-Inflammatory Drugs

**DOI:** 10.3389/fphar.2020.594427

**Published:** 2020-11-20

**Authors:** Raquel Jurado-Escobar, Inmaculada Doña, Gador Bogas-Herrera, Natalia Pérez-Sánchez, María Salas, José J. Laguna, Rosa Muñoz-Cano, Cristobalina Mayorga, María J. Torres, José A. Cornejo-García

**Affiliations:** ^1^ Allergy Research Group, Instituto de Investigación Biomédica de Málaga-IBIMA, Malaga, Spain; ^2^ Departamento de Medicina, Universidad de Málaga, Malaga, Spain; ^3^ Allergy Unit, Hospital Regional Universitario de Málaga, Malaga, Spain; ^4^ ARADyAL Network, Instituto de Salud Carlos III, Madrid, Spain; ^5^ Allergy Unit, Allergo-Anaesthesia Unit, Hospital Central de la Cruz Roja, Faculty of Medicine, Alfonso X El Sabio University, Madrid, Spain; ^6^ Allergy Section, Pneumology Department, Hospital Clinic, Universitat de Barcelona, Barcelona, Spain; ^7^ Nanostructures for Diagnosing and Treatment of Allergic Diseases Laboratory, Andalusian Center for Nanomedicine and Biotechnology-BIONAND, Malaga, Spain

**Keywords:** nonsteroidal anti-inflammatory drugs-hypersensitivity, cysteinyl-leukotrienes, transcellular metabolism, platelet-adherent leukocytes, integrins

## Abstract

Nonsteroidal anti-inflammatory drugs (NSAIDs) are among the most highly consumed drugs worldwide and the main triggers of drug hypersensitivity reactions. The most frequent reaction, named cross-reactive NSAID-hypersensitivity, is due to the pharmacological activity of these drugs by blocking the cyclooxygenase-1 enzyme. Such inhibition leads to cysteinyl-leukotriene synthesis, mainly LTE4, which are responsible for the reaction. Although the complete molecular picture of the underlying mechanisms remains elusive, the participation of platelet-adherent leukocytes (CD61^+^) and integrins have been described for NSAID-exacerbated respiratory disease (NERD). However, there is a lack of information concerning NSAID-induced urticaria/angioedema (NIUA), by far the most frequent clinical phenotype. Here we have evaluated the potential role of CD61^+^ leukocytes and integrins (CD18, CD11a, CD11b, and CD11c) in patients with NIUA, and included the other two phenotypes with cutaneous involvement, NSAID-exacerbated cutaneous disease (NECD) and blended reactions (simultaneous skin and airways involvement). A group NSAID-tolerant individuals was also included. During the acute phase of the reaction, the three clinical phenotypes showed increased frequencies of CD61^+^ neutrophils, eosinophils, and monocytes compared to controls, which correlated with urinary LTE4 levels. However, no correlation was found between these variables at basal state. Furthermore, increased expressions of CD18 and CD11a were found in the three CD61^+^ leukocytes subsets in NIUA, NECD and blended reactions during the acute phase when compared with CD61^−^leukocyte subpopulations. During the acute phase, CD61^+^ neutrophils, eosinophils and monocytes showed increased CD18 and CD11a expression when compared with CD61^+^ leukocytes at basal state. No differences were found when comparing controls and CD61^+^ leukocytes at basal state. Our results support the participation of platelet-adherent leukocytes and integrins in cutaneous cross-hypersensitivity to NSAIDs and provide a link between these cells and arachidonic acid metabolism. Our findings also suggest that these reactions do not involve a systemic imbalance in the frequency of CD61^+^ cells/integrin expression or levels of LTE4, which represents a substantial difference to NERD. Although further studies are needed, our results shed light on the molecular basis of cutaneous cross-reactive NSAID-hypersensitivity, providing potential targets for therapy through the inhibition of platelet-leukocyte interactions.

## Introduction

Nonsteroidal anti-inflammatory drugs (NSAIDs) are among the most highly consumed drugs worldwide because of their adequacy for treating pain and inflammatory processes (Fosbol et al., 2008; Conaghan, 2012; Duong et al., 2014). However, they are also responsible for 21–25% of adverse drug reactions, including drug hypersensitivity (Kowalski et al., 2011). The most frequent NSAID-hypersensitivity type belongs to the cross-reactive category, with patients reacting to NSAIDs from different chemical groups in the absence of specific immunological recognition (Dona et al., 2012; Dona et al., 2014; Dona et al., 2020).

Three cross-reactive clinical phenotypes have been recognized in the latest classification of NSAID-hypersensitivity by the European Academy of Allergy and Clinical Immunology; NSAID-exacerbated respiratory disease (NERD), in patients with rhinitis and/or asthma with or without nasal polyposis; NSAID-exacerbated cutaneous disease (NECD), in patients with underlying chronic spontaneous urticaria; and NSAID-induced urticaria/angioedema (NIUA), in otherwise healthy individuals (Kowalski et al., 2013). The latter is the most frequent clinical entity induced by drug hypersensitivity (Dona et al., 2014). Our group has recently described a frequent phenotype, blended reactions, with patients suffering from simultaneous cutaneous and respiratory involvement (Dona et al., 2018).

Concerning the underlying mechanisms, the precipitation of asthma attacks after acetylsalicylic acid (ASA) intake in NSAID-hypersensitive asthmatics was linked to cyclooxygenase (COX)-1 inhibition, and subsequent prostaglandin synthesis blockage. Such inhibition shunts the arachidonic acid (AA) metabolism toward pro-inflammatory cysteinyl-leukotrienes (CysLTs; LTC4, LTD4, and LTE4) biosynthesis, responsible for triggering a reaction in susceptible individuals (Szczeklik et al., 1975; Stevenson et al., 2001; Kowalski et al., 2019).

AA released from cellular membranes by cytosolic phospholipase A2 (cPLA2) is oxidized by 5-lipooxygenase (5-LO) to leukotriene (LT) A4 in inflammatory leukocytes (Reid et al., 1990). In monocytes, mast cells, eosinophils, and basophils LTA4 is conjugated to reduced glutathione by LTC4 synthase (LTC4S) to form LTC4. This is exported by the cell and enzymatically converted into LTD4, and then into the stable metabolite LTE4. In neutrophils, which lack LTC4S activity, LTA4 is hydrolyzed by LTA4 hydrolase (LTA4H) to form LTB4 (Lam et al., 1994).

This pathogenic model was initially proposed for NERD (Szczeklik et al., 1975), and supported by the presence of increased levels of CysLTs after ASA challenge (Szczeklik et al., 1996; Antczak et al., 2002; Swierczynska et al., 2003; Sanak et al., 2004; Gaber et al., 2008), and further extended to NECD (Mastalerz et al., 2004; Setkowicz et al., 2009). Lower baseline levels of PGE2 and increased values of CysLTs have been found in induced sputum from NERD when compared with ASA-tolerant asthmatics and chronic rhinosinusitis with nasal polyposis patients (Mastalerz et al., 2019). Additionally, PGE2 decreased and CysLTs increased after ASA challenge in NERD (Mastalerz et al., 2019), with significant differences compared with their basal values and with ASA-tolerant asthmatics. In both NIUA and NECD, we have recently reported increased LTE4 and 9a,11b-PGF2 levels after ASA challenge, which decreased at the basal state to values similar to those found in controls (Dona et al., 2019).

It is known that eosinophils, basophils, mast cells, and macrophages synthesize LTC4 but not how LTA4 is provided at sufficient quantities to produce the high basal levels of CysLTs described in NERD (Oosaki et al., 1997; Mita et al., 2001). Neutrophils have the highest 5-LO activity and their production of LTA4 exceeds their capacity to form LTB4 via LTA4H. The lack of LTC4S activity in neutrophils seems to be balanced by platelets, which possess abundant LTC4S activity in the absence of 5-LO (Penrose et al., 1995; Sala et al., 1999). In fact, *ex vivo* studies have shown that platelets can convert LTA4 from neutrophils or monocytes into LTC4 by a transcellular pathway that requires P-selectin-dependent interactions between platelets and leukocytes (Bigby and Meslier, 1989; Maclouf et al., 1994; Maugeri et al., 1994). Moreover, a key role of P-selectin-dependent platelets-leukocytes adherence have been described in an asthma mouse model of allergen-induced pulmonary eosinophilia and airway remodeling, which includes a subsequent augmentation of leukocyte integrin function (Pitchford et al., 2005). The underlying platelet-dependent pathway in this model of asthma requires the binding of platelet-associated P-selectin to leukocyte associated PSGL-1 (Pitchford et al., 2005). Such interaction primes leukocytes for adhesion to endothelial cells by up-regulating the expression and avidity of integrins, as it has been demonstrated in eosinophils, neutrophils, and monocytes (da Costa Martins et al., 2006; Xu et al., 2007; Johansson and Mosher, 2011). Concerning NSAID-hypersensitivity, a key role of platelet-adherent leukocytes and integrins (CD18, CD11a, CD11b, and CD11c) have been proposed for NERD (Laidlaw et al., 2012).

As platelet adherence to leukocytes permit the adhesion of both platelets and leukocytes to the endothelium, potentially increasing transcellular metabolism, alterations in platelet-leukocytes interactions may influence CysLTs production and trigger a cutaneous hypersensitivity reaction to NSAIDs, as reported for NERD (Laidlaw et al., 2012). However, despite its frequency, there is a lack of information concerning the role of platelet-leukocytes interactions in NIUA.

The aim of this work was to evaluate the potential participation of platelet-adherent leukocytes in NIUA, the most common phenotype in drug hypersensitivity. In addition, we have included a group of patients suffering from NECD and other with blended reactions, the other two phenotypes displaying cutaneous involvement.

## Methods

### Subjects

We included patients aged 18–60 years with a confirmed diagnosis of NSAID cross-reactive hypersensitivity who attended the Allergy Unit of the Malaga Regional University Hospital (Malaga, Spain) between March 2017 and February 2020.

Only patients reporting at least three episodes of acute urticaria, i.e., NIUA, exacerbation of their underlying chronic spontaneous urticaria, i.e., NECD, or blended reactions (skin and airways involvement) to NSAIDs were considered. Cross-reactive hypersensitivity was confirmed by a drug provocation test (DPT) with ASA.

We also included a control group of age and sex-matched individuals who reported regularly taking NSAIDs, including strong COX-1 inhibitors such as ASA and indomethacin, without developing a clinical reaction, and had no history of chronic spontaneous urticaria, drug hypersensitivity, rhinitis and/or asthma or nasal polyposis. A subset of these controls was also administered ASA.

All participants gave informed consent. The study was approved by the Ethics Committee of Malaga Regional University Hospital and conducted according to the principles of the Declaration of Helsinki.

### Oral Drug Provocation Test

ASA DPT was performed in a single-blind manner as reported previously (Dona et al., 2018), giving placebo capsules at different times on the first day. ASA and placebo were given in opaque capsules prepared by the hospital pharmacy service. Other medications were withheld before testing, in accordance with international guidelines (Dona et al., 2018).

For DPT to ASA, two doses were administered orally with an interval of 3 h (50 and 100 mg) on the second day. If negative, two larger doses of ASA (250 and 500 mg) were administered on the third day, with a 3 h interval. The procedure was stopped if cutaneous and/or respiratory symptoms or changes in vital signs (cardiac rhythm alterations, decrease in peak expiratory flow or hypotension) appeared, and symptoms were evaluated and treated (Dona et al., 2018). If no symptoms appeared during these periods, this was followed by a 2 days/8 h course of the therapeutic dose (500 mg) after a gap of 24 h (Dona et al., 2018).

### Flow Cytometry Analysis

Peripheral blood was collected in heparinised tubes from both patients and controls, and immediately assayed. For patients, a blood sample was obtained in the absence of clinical symptoms (basal state) and another one during the first half an hour after a positive DPT result (acute phase). For flow cytometry studies, in a subset of controls taking ASA, a blood sample was obtained before ASA intake and another during an hour after intake, whereas for the rest of controls blood samples were obtained at the moment of their enrollment in the study.

One hundred microliters of whole blood were directly incubated with specific antibodies for CD45, CD16, CCR3 (CD193), CD61, CD11a, CD11b, CD11c, P-selectin glycoprotein ligand 1 (PSLG-1; CD162), and/or CD18, or adequate isotype controls (BioLegend) for 20 min. After erythrocyte lysis and washing, at least 20,000 CD45^+^ cells were obtained in a FACSCanto cytometer (BD Biosciences), and analyzed with the FlowJo software Version 10.6 (TreeStar). According to their side scatter characteristics, CD45^+^ leukocytes were classified as granulocytes, monocytes, or lymphocytes. In addition to their side scatter properties, neutrophils and eosinophils were further defined from the granulocyte population by the expression of CD16 or CCR3 (Supplementary Figure S1). All these populations were assessed for the presence of adherent platelets by the expression of CD61. Finally, in both platelet-adherent and platelet-nonadherent subsets, adhesion markers were determined through their mean fluorescence intensity (MFI).

### LTE4 Determination

Patient urine samples were collected at basal state, that is, before challenge, and within the first 3 h after a positive challenge as described (Dona et al., 2019). One urine sample was also obtained from controls regularly taking NSAIDs. LTE4 was determined by high-perfomance liquid chromatography-tandem mass spectrometry, and results were expressed in pg/mg of creatinine (Dona et al., 2019).

### Statistical Analysis

Descriptive statistics (frequency, mean, and SD) were used to summarize data. Comparison between groups were performed using the Kruskal-Wallis test followed by Mann-Whitney when necessary, and related samples were evaluated with the Wilcoxon test. Correlation between variables was estimated with the Pearson correlation coefficient. All analyses were performed with GraphPad version 7.04 for Windows (GrapPad Software, La Jolla, CA, United States). All *p*-values ≤ 0.05 were considered statistically significant.

## Results

### Demographic and Clinical Data

We finally included a total of 59 patients and 19 controls. Patients were classified as having NIUA (*n* = 35), NECD (*n* = 14) or blended reactions (*n* = 10). A subset of controls was administered ASA (*n* = 10). The distribution of individuals between groups is shown in Table 1. None of the patients in the blended reactions group suffered from nasal polyposis. No significant differences in sex were found between patients and controls (*p* = 0.926). In addition, although patients with blended reactions showed a higher median age, no statistically significant differences were found between the groups (*p* = 0.082) (Table 1).

**TABLE 1:** Demographic and clinical data for patients and controls.

	Controls (*n* = 19)	NIUA (*n* = 35)	NECD (*n* = 14)	Blended (*n* = 10)	*p*-value
Sex (female/male)	10/9	20/15	9/5	6/5	0.926
Age, median (range)	37 (33.2–45)	40.5 (31.5–48)	41 (30.2–50)	53.5 (43.7–60)	0.082
Acetylsalicylic acid cumulative dose	NA	355 ± 312.9	352.9 ± 265.7	232 ± 186.8	0.710
Time interval	NA	54 ± 34.3	75.7 ± 43.9	28 ± 19.2	0.082

NA, not applicable; NIUA, NSAID-induced acute urticaria/angioedema; NECD, NSAID-exacerbated cutaneous disease.

Concerning the cumulative ASA dose that elicited a reaction during this procedure, no significant differences were found between the three groups of patients (*p* = 0.710), although the lowest dose was found for those with blended reactions (Table 1). Finally, no significant differences were found between the three NSAID-hypersensitive groups of patients regarding the time interval elapsed between the last dose administered via DPT and the appearance of clinical symptoms (*p* = 0.082), although the lowest interval corresponded to blended reactions (Table 1).

### Platelet-Adherent Leukocytes

We evaluated the presence of platelet-adherent leukocytes in whole blood by flow cytometry using the protein tyrosine phosphatase CD45, which is a pan-leukocyte antigen. Positive CD45 cells were further grouped into different categories based only on their specific light side scatter characteristics (monocytes and lymphocytes) or also considering the expression of CD16 or CCR3 (neutrophils and eosinophils, respectively) (Supplementary Figure S1). Platelet-adhesion was determined through the CD61 antigen, which is an integrin expressed in platelets (Pitchford et al., 2005). Our preliminary results did not find any differences between in the ASA-controls group before and after ASA intake for any of the variables analyzed (data not shown), therefore ASA administration in controls was not considered necessary for subsequent comparisons.

**FIGURE 1:**
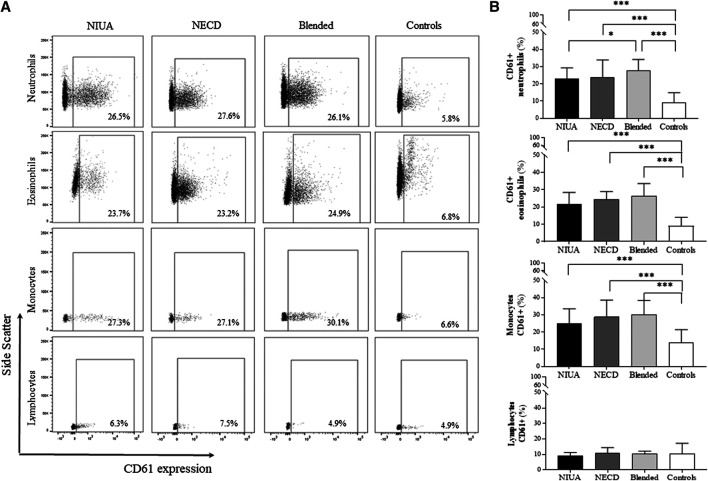
Platelet-adherent leukocytes in peripheral blood during the acute phase in NIUA, NECD or blended reactions, and from controls. **(A)** Representative histograms of platelet-adherent neutrophils [identified as CD45^+^CD16^+^ cells in the granulocyte side scatter (SSC) gate], eosinophils (CD45 + CCR3+ in the granulocyte SSC gate), monocytes (CD45^+^ in the monocyte SSC gate), and lymphocytes (CD45^+^ in the lymphocyte SSC gate). The percentage of each cell type with adherent platelets is presented. **(B)** Percentages of platelet-adherent leukocytes (CD61^+^) in patients during the acute phase and controls. Data are expressed as mean ± SD (*p ≤ 0.05; **p ≤ 0.001; ***p < 0.0001).

We detected the presence of platelet-adherent leukocytes in both patients and controls (Figure 1A). During the acute phase, i.e., after a positive challenge result, CD61^+^ neutrophils were more frequent in NIUA, NECD, and blended reactions than in controls (*p* < 0.0001 for all comparisons) (Figure 1B). Similar results were also found when evaluated CD61^+^ eosinophils and CD61^+^ monocytes in all groups of patients respect to control individuals (*p* < 0.0001 for all comparisons) (Figure 1B). In addition, CD61^+^ neutrophils were also increased in blended reactions when compared with NIUA (*p* = 0.034). No statistically significant differences were found between any of the groups regarding CD61^+^ lymphocytes (Figure 1B).

We further analyzed if there were differences in the percentage of platelet-adherent leukocytes between the acute phase and the basal state (Table 2). Such percentage significantly decreased when compared these two time points in the three groups of patients in neutrophils (22.9 ± 6.6 vs. 8.9 ± 4.3 in NIUA, *p* < 0.001; 23 ± 9.1 vs. 9.4 ± 4.2 in NECD, *p* = 0.003; and 28.4 ± 6.5 vs. 10.2 ± 5.4 in blended reactions, *p* = 0.008); eosinophils (21.8 ± 6.8 vs. 14.8 ± 8 in NIUA, *p* = 0.001; 24.2 ± 4.4 vs. 11.3 ± 5.4 in NECD, *p* = 0.003; and 27.1 ± 7.3 vs. 12.2 ± 6.2 in blended reactions, *p* = 0.011), and monocytes (24.3 ± 8.3 vs. 11.9 ± 6.4 in NIUA, *p* < 0.001; 27.4 ± 11.1 vs. 12.3 ± 4.2 in NECD, *p* = 0.003; and 30.8 ± 8.6 vs. 12.6 ± 8.2 in blended reactions, *p* = 0.008) (Table 2). No statistically significant differences were found between these two states in lymphocytes for any of the groups considered (Supplementary Figure S2).

**TABLE 2:** Platelet-adherent leukocytes in the different phenotypes of cross-hypersensitivity to NSAIDs during the acute phase and the basal state.

		% CD61^+^ (mean ± SD)			
		Neutrophils	Eosinophils	Monocytes	Lymphocytes
NIUA	Acute	22.9 ± 6.6	21.8 ± 6.8	24.3 ± 8.3	8.8 ± 2.7
	Basal	8.9 ± 4.3	14.8 ± 8	11.9 ± 6.4	9.7 ± 5.2
	*p*-value	<0.001	0.001	<0.001	0.489
NECD	Acute	23 ± 9.1	24.2 ± 4.4	27.4 ± 11.1	10.8 ± 2.7
	Basal	9.4 ± 4.2	11.3 ± 5.4	12.3 ± 4.2	10.3 ± 4.1
	*p*-value	0.003	0.003	0.003	0.807
Blended	Acute	28.4 ± 6.5	27.1 ± 7.3	30.8 ± 8.6	10.3 ± 1.8
	Basal	10.2 ± 5.4	12.2 ± 6.2	12.6 ± 8.2	9.1 ± 6.4
	*p*-value	0.008	0.011	0.008	0.859

NIUA, NSAID-induced acute urticaria/angioedema; NECD, NSAID-exacerbated cutaneous disease.

### Expression of Integrins

**FIGURE 2:**
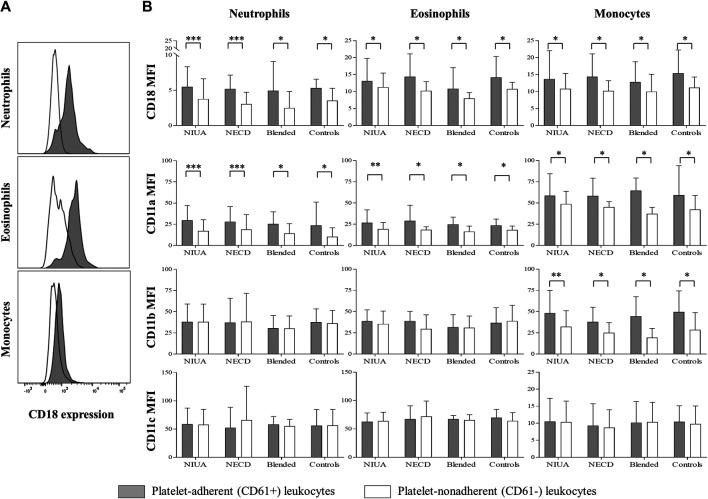
**A)** Representative histograms of relative CD18 expression by platelet-adherent (CD61^+^; solid gray) and platelet-nonadherent (CD61^−^; white line) leukocytes. **(B)** Relative expression of CD18, CD11a, CD11b, and CD11c integrins by platelet-adherent (CD61^+^) and platelet-nonadherent (CD61^−^) leukocytes subpopulations during the acute phase in NSAID-induced urticaria/angioedema (NIUA), NSAID-exacerbated cutaneous disease (NECD) or blended reactions, and in controls. Data are expressed as mean ± SD (*p ≤ 0.05; **p ≤ 0.001; ***p < 0.0001).

MFI of CD18, CD11a, CD11b, and CD11b for both platelet-adherent and platelet-nonadherent leukocytes (CD61^+^ and CD61^−^, respectively) during the acute phase are shown in Figure 2. We found a statistically significant increased expression in CD18 and CD11a in neutrophils, eosinophils and monocytes in the CD61^+^ subset in the three groups of cross-hypersensitive patients during the acute phase. Such increase was also detected in the control group (Figure 2). Concerning CD11b, we only found a statistically significant increase in platelet-adherent monocytes. Regarding CD11c, no differences were found for any of these three leukocyte populations in patients and controls when compared the CD61^+^ and CD61^−^populations. Finally, there were no differences between the platelet-adherent and platelet-nonadherent lymphocytes for any of the integrins evaluated in patients and controls (Supplementary Figure S2).

We also explored potential changes in integrin expression in the CD61^+^ subset between the acute phase and the basal state in the four cell types considered. MFI of CD18 and CD11a were significantly increased during the acute phase in neutrophils, eosinophils and monocytes for NIUA, NECD and blended reactions, with no differences found in lymphocytes. CD11b and CD11c did not show expression changes when compared the acute phase and the basal state for any of the group of patients included (Table 3).

**TABLE 3:** CD61^+^ leukocyte expression of the integrins CD18, CD11a, CD11b, and CD11c in the different phenotypes of cross-hypersensitivity to NSAIDs during the acute phase and the basal state.

		NSAID-induced urticaria/angioedema			NSAID-exacerbated cutaneous disease			Blended		
Integrins	Leukocytes	Acute	Basal	*p*-value	Acute	Basal	*p*-value	Acute	Basal	*p*-value
CD18	Neutrophils	5.3 ± 2.7	3.7 ± 1.2	0.006	5.3 ± 2.3	3.5 ± 1.1	0.011	4.8 ± 4.1	2.2 ± 0.4	0.021
	Eosinophils	12.8 ± 6.5	9.6 ± 3.6	0.023	12.5 ± 6.8	5.6 ± 2.1	0.003	10.1 ± 4.6	3.5 ± 1.8	0.015
	Monocytes	13.4 ± 8.3	9.5 ± 3.4	0.005	14.7 ± 7.1	3.8 ± 1.2	0.001	12.4 ± 5.2	4.9 ± 1.3	0.018
	Lymphocytes	10.6 ± 4.4	9.8 ± 3.3	0.427	10.3 ± 3.6	8.6 ± 2.9	0.249	11.8 ± 4.2	10.22 ± 4.2	0.310
CD11a	Neutrophils	30 ± 17.7	20.5 ± 3.8	0.018	28.2 ± 17.8	17.9 ± 3.3	0.016	22.1 ± 11.1	14.1 ± 3.9	0.038
	Eosinophils	25.8 ± 14.3	18.4 ± 4.9	0.036	32.3 ± 19.5	16.4 ± 5	0.013	22.7 ± 8.3	11.8 ± 2.9	0.008
	Monocytes	57.7 ± 25.7	42.4 ± 11.4	0.011	60.3 ± 22.5	17.3 ± 5.1	0.001	63.4 ± 14.2	21.2 ± 4.3	0.018
	Lymphocytes	43 ± 20.1	42.5 ± 16.3	0.993	49.6 ± 27.5	35.8 ± 9.6	0.279	45.7 ± 11.8	45.9 ± 9.6	0.176
CD11b	Neutrophils	37.8 ± 21.3	43.4 ± 49.7	0.533	39.5 ± 28	27.1 ± 37.2	0.101	27.7 ± 15.2	31.6 ± 44.1	0.374
	Eosinophils	39.3 ± 12.5	37.5 ± 10.7	0.317	38.1 ± 12.3	29.4 ± 33.4	0.133	33.4 ± 13.3	33.9 ± 39.9	0.441
	Monocytes	49.2 ± 26.4	42.2 ± 14.2	0.235	37.6 ± 17.6	26.5 ± 28.8	0.101	36.8 ± 16.9	34.8 ± 32	0.441
	Lymphocytes	10.7 ± 6.8	11.5 ± 6.1	0.412	9.2 ± 6.5	11.6 ± 8.7	0.463	9.9 ± 6.3	9.5 ± 11.1	0.374
CD11c	Neutrophils	59.7 ± 28.3	49.1 ± 25.6	0.104	52.1 ± 36.4	49.1 ± 16.1	0.861	56.3 ± 16.7	48.8 ± 21.9	0.265
	Eosinophils	62.6 ± 15.9	62.1 ± 14.8	0.837	67.3 ± 23.4	65.1 ± 14.1	0.917	66.2 ± 7.2	63.3 ± 12.8	0.515
	Monocytes	37.7 ± 22.6	31.7 ± 21.4	0.238	41.9 ± 30.6	25.9 ± 13.8	0.196	29.8 ± 10.8	28.9 ± 4.6	0.859
	Lymphocytes	7.9 ± 6	9.9 ± 12.5	0.688	7.3 ± 8.4	5 ± 3.4	0.701	9.6 ± 4.6	9.9 ± 12.6	0.261

Finally, NIUA, NECD, and blended reactions showed a similar pattern in the expression levels of PSLG-1 in all leukocytes subsets, with no differences between the acute phase and the basal state (Supplementary Figure S3).

### LTE4 Levels and Platelet-Adherent Leukocytes

**FIGURE 3:**
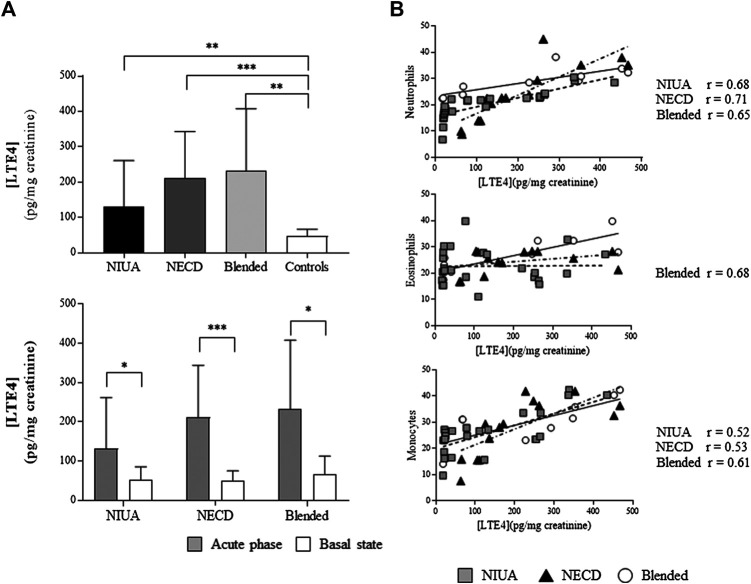
Platelet-adherent leukocytes and urinary LTE4. **(A)** LTE4 levels in patients with NSAID-induced urticaria/angioedema (NIUA), NSAID-exacerbated cutaneous disease (NECD), or blended reactions during the acute phase, and controls (**top**), and comparisons between the acute phase and the basal state in patients (**bottom**). **(B)** LTE4 in the three clinical entities induced by NSAID-hypersensitivity vs. the percentage of platelet-adherent neutrophils (**top**), eosinophils (**middle**), and monocytes (**bottom**) in peripheral blood. Data are expressed as mean ± SD (*p ≤ 0.05; **p ≤ 0.001; ***p < 0.0001). The value of r represents the effect size, determined by the Pearson correlation coefficient. Gray squares, NIUA; black triangles, NECD; white circles, blended reactions.

We determined urinary LTE4 levels during the acute phase and in the basal state in all patients with NECD or blended reactions as well as in a subset of NIUA patients (*n* = 24) and controls (*n* = 17). Urinary LTE4 levels during the acute phase were increased when compared with controls in the three clinical phenotypes: NIUA (*p* = 0.01), NECD (*p* < 0.0001) and blended reactions (*p* = 0.0002) (Figure 3A, top). In addition, these levels were also increased when compared the acute phase with the basal state (*p* = 0.045 for NIUA, *p* = 0.0006 for NECD, and *p* = 0.001 for blended reactions) (Figure 3A, bottom). We did not find differences between urinary LTE levels when compared the basal state of the three groups of patients with those from the control group (data not shown).

We also evaluated the potential correlation between urinary LTE levels and platelet-adherent leukocytes during the acute phase. We found a positive correlation between this variable and platelet adherent-neutrophils in NIUA (*r* = 0.68, *p* < 0.0001), NECD (*r* = 0.71, *p* = 0.0001), and blended reactions (*r* = 0.65, *p* = 0.005). Concerning platelet-adherent eosinophils, statistically significant correlation was found only for blended reactions (*r* = 0.68, *p* = 0.003). Moderate correlations were also found with platelet-adherent monocytes in NIUA (*r* = 0.52, *p* < 0.001), NECD (*r* = 0.53, *p* = 0.003), and blended reactions (*r* = 0.6, *p* = 0.008). No correlations were detected regarding urinary LTE levels and platelet-adherent lymphocytes. Besides, no correlations were found between these levels and platelet-adherent leukocytes during the basal state (data not shown).

## Discussion

NSAIDs are widely accepted to be the main cause of drug hypersensitivity reactions, and NIUA the most frequent phenotype. In addition to NIUA, two other clinical entities induced by cross-reactive hypersensitivity to NSAIDs show cutaneous symptoms, i.e., NECD and blended reactions. The underlying mechanism in cross-reactive hypersensitivity, initially proposed for NERD, involves the pharmacological inhibition of COX-1 by NSAIDs, blocking prostaglandins synthesis and shunting the AA metabolism toward CysLTs production (Szczeklik et al., 1975; Szczeklik et al., 1996). This mechanism has been supported by multiple studies (Antczak et al., 2002; Swierczynska et al., 2003; Sanak et al., 2004; Gaber et al., 2008), and further extended to NECD and NIUA (Dona et al., 2020).

Although in more than 70% of patients with blended reactions diagnosis can be achieved by nasal provocation test with Lys-ASA (Dona et al., 2018), here we have included only blended patients with a positive oral DPT to ASA to avoid potential differences in the intensity of the stimulus due to the administration route. Here we have showed for the first time that, after a positive DPT with ASA, patients with blended reactions also showed an increase in urinary LTE4 compared with their basal state, with no differences between basal state levels and those of controls. These results highlight an important difference between blended reactions and NERD for which high baseline LTE4 concentrations have been repeatedly reported (Christie et al., 1991; Kumlin et al., 1992; Oosaki et al., 1997; Higashi et al., 2002; Gaber et al., 2008; Higashi et al., 2010). Such difference may be a reflection of the underlying respiratory disease, as none of patients we labeled as blended presented nasal polyposis in their medical history (Bochenek et al., 2018). Blended reactions represent a heterogeneous group of entities which include cutaneous (urticaria/angioedema) and respiratory symptoms (rhinitis/asthma with or without nasal polyposis); cutaneous symptoms and glottis edema; cutaneous and respiratory symptoms accompanied with glottis edema; and a combination of cutaneous, respiratory and gastrointestinal symptoms (abdominal pain, diarrhea, nausea, vomiting) (Dona et al., 2018). Moreover, we cannot rule out that other phenotypes could be further included in this category (Dona et al., 2020), as described for asthma (Mastalerz et al., 2015) and NERD (Celejewska-Wojcik et al., 2020).

Despite of the participation of COX-1 inhibition and CysLTs in NSAID-hypersensitivity, the molecular basis of these reactions remains elusive. In addition to their inflammatory role in cardiovascular disease (Massberg et al., 2002) and allergen response in bronchial asthma (Moritani et al., 1998), platelet-adherent leukocytes have been shown to play a key role in NERD, as well as integrin subunits expression (Laidlaw et al., 2012). In fact, the frequency of CD61^+^ neutrophils, eosinophils, and monocytes are increased in NERD compared with controls, and these frequencies are correlated with systemic LTE4 levels (Laidlaw et al., 2012).

We have also found that CD61^+^ leukocyte levels increase in the three groups of patients with cutaneous symptoms induced by cross-reactive NSAID-hypersensitivity after a positive DPT to ASA (acute phase) (Figure 1). However, we did not observe any increase in the frequency of CD61^+^ leukocytes in any group of patients at the basal state compared to controls. In addition to dermal edema, the classic histopathological description of urticaria also includes a sparse perivascular infiltrate composed of neutrophils, eosinophils, macrophages and lymphocytes (Zuberbier et al., 2009), although some subgroups of urticaria may exist according to the predominance of neutrophils and lymphocytes (Barzilai et al., 2017). As proposed for NERD (Pitchford et al., 2005), platelets may prime leukocyte adhesion to the endothelium and amplify cutaneous inflammation during a hypersensitivity reaction to NSAIDs as a consequence of a pathogenic change in the homeostasis of this system. In fact, altered platelet function has been described in severe food-associated respiratory allergy (Obeso et al., 2018), and changes in platelet-related genes have been described in some types of chronic spontaneous urticaria (Gimenez-Arnau et al., 2017). Moreover, platelets have been associated with the etiology of a wide range of pathologies behind coagulation disorders (Gianazza et al., 2020), and some of platelet-related compounds may represent potential biomarkers (Duarte et al., 2013; Eguiluz-Gracia et al., 2018; Liao et al., 2018; Liao et al., 2020; Sokolowska et al., 2020).

Interestingly, we have also found that the percentage of CD61^+^ leukocytes correlated with urinary LTE4 levels (neutrophils and monocytes in the three phenotypes, and eosinophils in blended reactions) during the acute phase (Figure 3). Nevertheless, no correlation was found between the frequencies of CD61^+^ leukocytes and LTE4 levels at the basal state. In addition to the lack of differences in LTE4 levels between blended reactions in the basal state and controls described here, we have previously reported that differences do not existed between LTE4 basal levels in NIUA and NECD and LTE4 levels in controls (Dona et al., 2019). Although COX-1 inhibition and the dysregulation of LTE4 synthesis is thought to be shared by the different clinical entities induced by cross-reactive NSAID-hypersensitivity, our results suggest that a specific pattern exists for NERD and another one for the other three phenotypes as systemic LTE4 production does not exist in NIUA, NECD or blended reactions in our studies.

As adhesion to the endothelium has been reported to require up-regulation of integrins in neutrophils (Xu et al., 2007), eosinophils (Johansson and Mosher, 2011), and monocytes (da Costa Martins et al., 2006), we have also explored their expression in NIUA, NECD and blended reactions. As for NERD, we did not find any differences in PSGL-1 expression in our study. During the acute phase, CD18 and CD11a were significantly increased in CD61^+^ leukocytes compared to CD61^−^leukocytes in all patient groups, whereas CD11b was increased only in monocytes (Figure 2). We also found that CD18 and CD11a expression were significantly elevated in CD61^+^ cells when in the acute phase compared to the basal state (Table 3). CD18 interacts with the other molecules to form β2 integrins in order to adhere leukocytes to endothelial and epithelial cells. Our results agree in general with those obtained for NERD (Laidlaw et al., 2012); however, we did not find any difference between CD61^+^ and CD61^−^leukocytes when evaluating integrin expression at the basal state (Supplementary Figure S2). These results agree with our previous findings reporting no increases in the frequencies of CD61^+^ leukocytes at the basal state compared to control samples, as well as the lack of correlation with urinary LTE levels in such state. Unlike NERD, in the other three phenotypes induced by cross-hypersensitivity to NSAIDs there is no systemic imbalance for AA metabolism or in platelet-leukocytes interaction homeostasis.

In summary, we found that platelet-adherent leukocytes and integrin expression are increased in cutaneous cross-reactive NSAID-hypersensitivity, suggesting that a potential imbalance in the interaction of these leukocytes and endothelial and/or epithelial cells may participate in the underlying pathogenic mechanism, as suggested for NERD (Laidlaw et al., 2012). Primed platelets may bind to leukocytes before their migration to the skin and modify the homeostasis of this process. Platelet priming may be triggered by the inhibition of PGE2 synthesis due to COX-1 blockade as it is known that this prostaglandin usually increases the threshold for platelet activation (van der Meijden and Heemskerk, 2019). Although further studies are needed, which should include affected skin and isolated platelets for functional analyses, our results shed light on the molecular basis of non-immunological, cutaneous hypersensitivity to NSAIDs and open new treatment possibilities through the potential inhibition of platelet-leukocytes interactions.

## Data Availability Statement

The raw data supporting the conclusions of this article will be made available by the authors, without undue reservation.

## Ethics Statement

The studies involving human participants were reviewed and approved by Ethics Committee of Malaga Regional University Hospital. The patients/participants provided their written informed consent to participate in this study.

## Author Contributions

ID and JAC-G designed the study. ID, NP-S, GB-H, MS, and MT recruited, evaluated and diagnosed patients. JL and RM-C revised all clinical data. RJ-E performed experiments, data analysis and drafted the article, and was supervised by JAC-G. CM and MT revised the article. ID and JAC-G are responsible for the final version. All authors revised and approved the submitted version of the article.

## Funding

This work was supported by Instituto de Salud Carlos III (ISCIII, Spanish Ministry of Science and Innovation) co-founded by Fondo Europeo de Desarrollo Regional-FEDER for Research Projects (PI17/01,593), the Thematic Networks and Co-operative Research Centers: ARADyAL RD16/0006/0001, 0007, and 0033, and from the Sociedad Española de Alergología e Inmunología Clínica (SEAIC; Ref. Convocatoria Ayudas 2016 and Convocatoria Ayudas 2018 Ref. 18 B02).

## Conflict of Interest

The authors declare that the research was conducted in the absence of any commercial or financial relationships that could be construed as a potential conflict of interest.
