# Supplementary material for: Platelet-Adherent Leukocytes Associated With Cutaneous Cross-Reactive Hypersensitivity to Nonsteroidal Anti-Inflammatory Drugs
Source: Front Pharmacol. 2020 Nov 20;11:594427. doi: 10.3389/fphar.2020.594427 (PMC7919189; doi:10.3389/fphar.2020.594427)
Supplement: Supplementary file 1 [file Presentation1_v1.PPTX]

## Slide 1
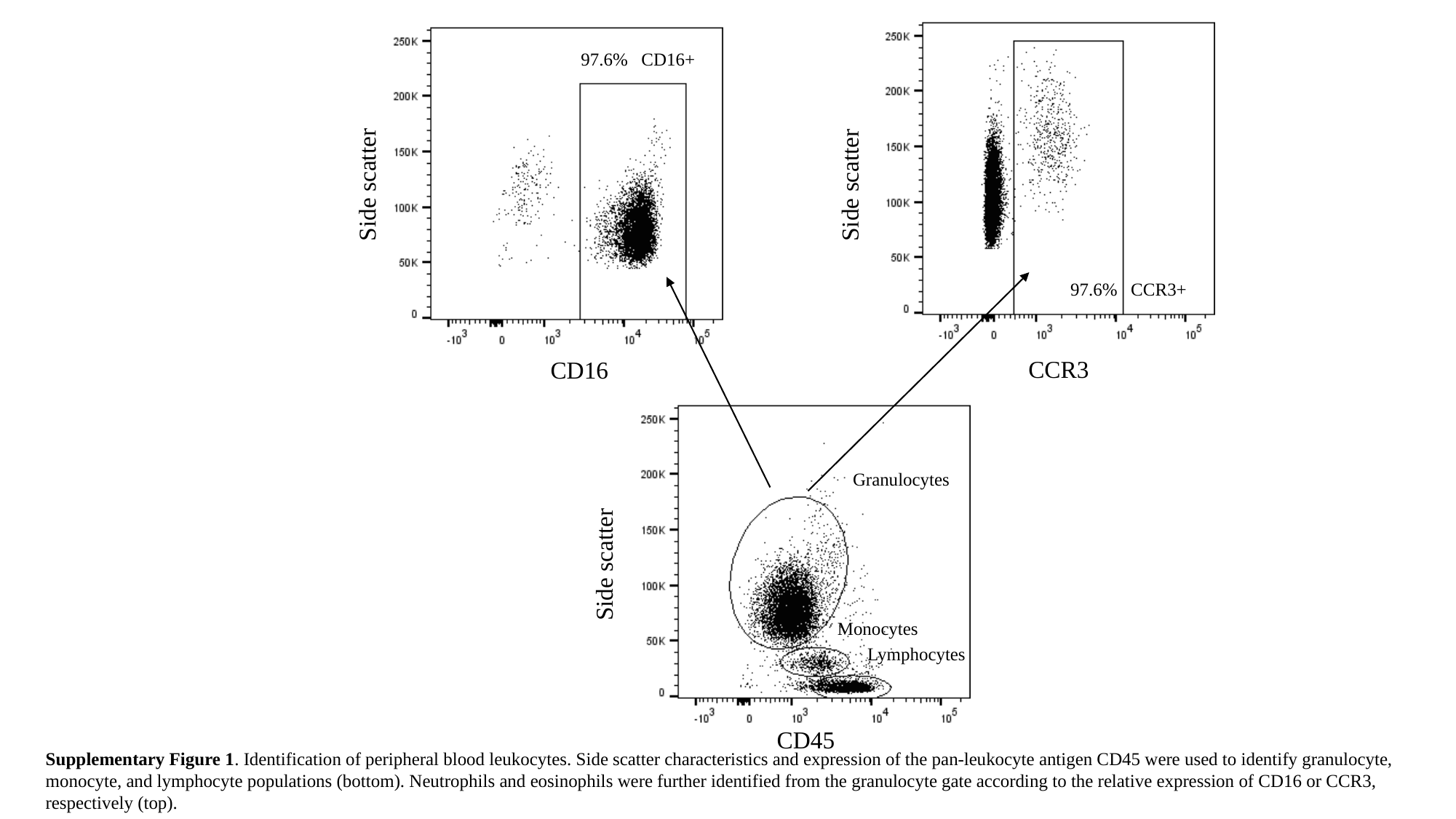

97.6% CD16+
Side scatter
Side scatter
97.6% CCR3+
CCR3
CD16
Granulocytes
Side scatter
Monocytes
Lymphocytes
CD45
Supplementary Figure 1. Identification of peripheral blood leukocytes. Side scatter characteristics and expression of the pan-leukocyte antigen CD45 were used to identify granulocyte, monocyte, and lymphocyte populations (bottom). Neutrophils and eosinophils were further identified from the granulocyte gate according to the relative expression of CD16 or CCR3, respectively (top).

## Slide 2
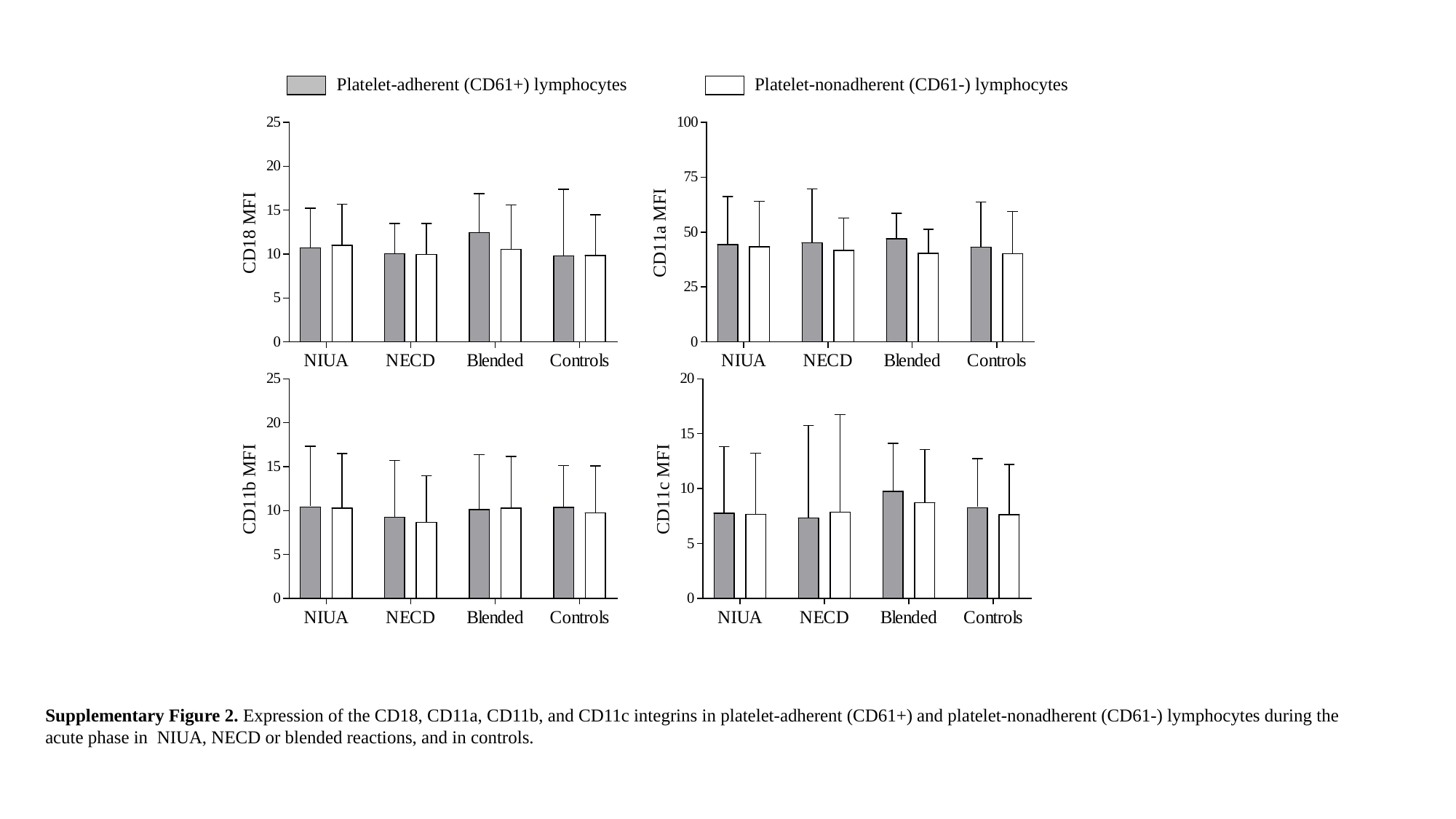

Platelet-adherent (CD61+) lymphocytes
Platelet-nonadherent (CD61-) lymphocytes
Supplementary Figure 2. Expression of the CD18, CD11a, CD11b, and CD11c integrins in platelet-adherent (CD61+) and platelet-nonadherent (CD61-) lymphocytes during the acute phase in NIUA, NECD or blended reactions, and in controls.

## Slide 3
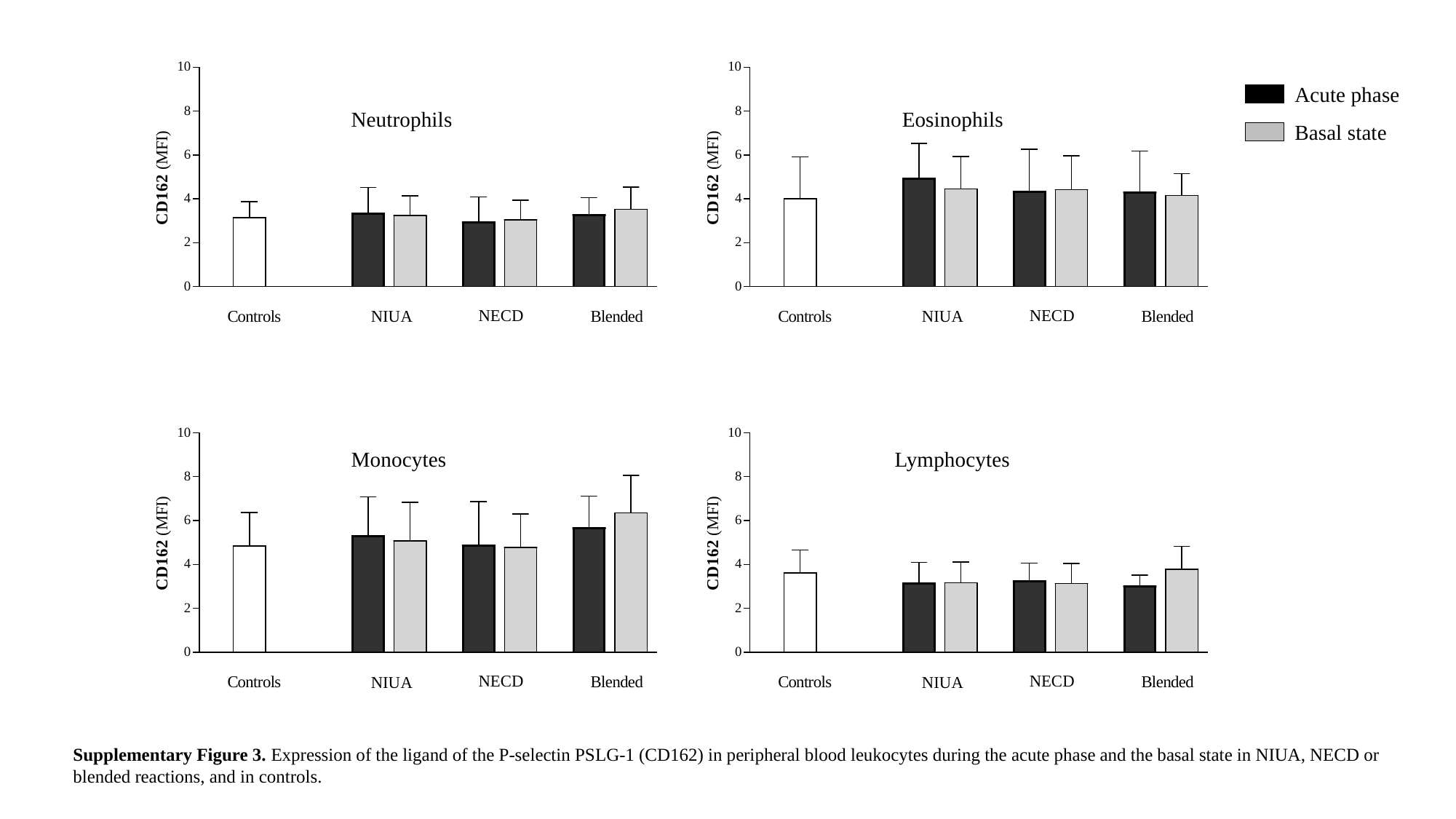

Neutrophils
Eosinophils
Monocytes
Lymphocytes
Acute phase
Basal state
Supplementary Figure 3. Expression of the ligand of the P-selectin PSLG-1 (CD162) in peripheral blood leukocytes during the acute phase and the basal state in NIUA, NECD or blended reactions, and in controls.
